# Factors affecting equitable access and uptake of COVID-19 vaccines in Ghana: a scoping review

**DOI:** 10.3389/fpubh.2025.1610765

**Published:** 2026-02-16

**Authors:** James Akazili, Dominic Anaseba, Samuel Chatio, Michel Adurayi Amenah, Daniel Malik Achala, Senait Aleamyehu Beshah, Chijioke O. Nwosu, Nyasha Masuka, John Thato Tlhakanelo, Ifeanyi Chikezie, Elizabeth Naa Adukwei Adote, Grace Njeri Muriithi, John Ele-Ojo Ataguba

**Affiliations:** 1 School of Public Health, C. K. Tedam University of Technology and Applied Sciences, Navrongo, Ghana; 2 Bergen Centre for Ethics and Priority, University of Bergen, Bergen, Norway; 3 Faculty of Public Health, Ghana College of Physicians and Surgeons, Accra, Ghana; 4 Navrongo Health Research Centre, Navrongo, Ghana; 5 African Health Economics and Policy Association (AfHEA), Accra, Ghana; 6 Ethiopian Public Health Institute, Addis Ababa, Ethiopia; 7 Department of Economics and Finance, University of the Free State, Bloemfontein, South Africa; 8 Zimbabwe College of Public Health Physicians, Harare, Zimbabwe; 9 Department of Family Medicine and Public Health, Faculty of Medicine, University of Botswana, Gaborone, Botswana; 10 Health Systems and Development Research Group, Veritas University Abuja, Abuja, Nigeria; 11 Department of Community Health Sciences, Max Rady College of Medicine, Winnipeg, MB, Canada; 12 Faculty of Health Sciences, University of Manitoba, Winnipeg, MB, Canada; 13 Partnership for Economic Policy (PEP), Nairobi, Kenya; 14 School of Health Systems and Public Health, University of Pretoria, Pretoria, South Africa

**Keywords:** coronavirus, vaccination, vaccine, equitable, access, uptake, scoping review, Ghana

## Abstract

**Background:**

The coronavirus disease (COVID-19) emerged as one of the most serious pandemics that impacted health systems and world economies. Vaccination against the pandemic was considered as an effective tool for the prevention and containment of the virus. Following the outbreak of the Coronavirus pandemic, efforts were made to enhance procurement and distribution of vaccines across countries with the view to containing the pandemic. However, evidence suggested that several factors hindered access, acceptance and use of the COVID-19 vaccines across the globe. This scoping review, thus, explored factors that influenced access, acceptance and use of the COVID-19 vaccines among Ghanaians and strategies that were needed to improve vaccine uptake especially for the vulnerable populations.

**Methods:**

We adopted the five-stage analytic framework developed by Arksey and O’Malley to map existing literature on what has been done and documented on the subject. We searched various electronic databases such as PubMed, Cochrane, African journal online (AJOL), and Google Scholar for relevant articles for the review.

**Results:**

In all, fifty-four (54) articles retrieved met our eligibility criteria and were included in this review. Health system factors including untimely payment of vaccinators allowances, shortfalls in logistics and vaccines, lack of transport and long queues at vaccination centers affected access and uptake of the COVID-19 vaccines in Ghana. Additionally, beliefs and perceptions including myths, misconceptions and misinformation around the virus and the vaccines affected people’s decision-making to participate in the vaccination exercise. Also, negative reportage through social media platforms created mistrust in COVID-19 vaccine intensions.

**Conclusion:**

Even though Ghana made significant progress in addressing the Coronavirus pandemic, hesitancy factors played a crucial role in diminishing Ghana’s effort towards meeting global targets in containing the virus and reducing its impact. Strengthening Ghana’s public health preparedness and response strategy, through a community-based approach and multi-stakeholder engagement, could improve immunization programs and vaccines uptake in addressing future pandemics.

## 1 Introduction

The coronavirus disease (COVID-19) since its emergence became one of the world’s most serious pandemics, that affected health systems and economies worldwide (1–4). The impact of the Coronavirus pandemic attracted the world’s attention leading a quick response in the mobilization of financial and technical resources to combat the pandemic (5). The pandemic had serious impact on individuals who were affected, their families and national economies (6). Low- and middle-income countries including Ghana, with already struggling healthcare systems in terms of inadequate human resources and logistical challenges were particularly affected (7), and the vulnerable population were the hardest hit by the COVID-19 pandemic (6). Vaccination became an eminent and effective strategy to mitigate the effect of the COVID-19 virus and maintain population health (8–13). However, this could only be achieved with high vaccination coverage (14).

As countries began to develop vaccines to tackle the COVID-19 pandemic, it was expected that these vaccines would be distributed widely across countries and used to reduce the spread and impact of the virus (15). This was, however, far from the reality as vaccine nationalism became a reality with high income countries (HICs) procuring more vaccines directly from manufacturers and hoarding them. This was in utter contravention of the COVAX initiative, a program which was initiated to bridge access gap and ensure equity in the delivery of the Coronavirus vaccines across countries (16). This practice propagated vaccine inequity and limited access to vaccines especially for lower- and middle-income countries (LMICs) during the peak of the pandemic (16). The net effect of this was that majority of potential eligible candidates for the COVID-19 vaccination, lost the opportunity as only 10% of Africa’s population was fully vaccinated as of January 2022 (17) compared to 75% of Europeans, 63% of North Americans and 85% in the Oceania around the same period (18).

In Ghana, only 15.9% of the population was fully vaccinated as of April 2022 (19). Several studies in Ghana cited various reasons including perceived side effects and doubt about the efficacy of the COVID-19 vaccines as the cause of the low coverage of the vaccines in the country (20).

This scoping review, therefore, sought to explore and gain deeper insights on what has been documented, the lessons Coronavirus pandemic brought to the world especially for developing countries such as Ghana so as to guide and strengthen national emergency preparedness for combating future vaccine preventable pandemics. We specifically sought to assess equitable and timely access to and uptake of the COVID-19 vaccines among the general population as well as the different population groups; barriers to access and uptake of the COVID-19 vaccines; and measures that could be taken to improve future access and uptake of vaccines especially for the vulnerable population in Ghana. The review, thus, explored the following research questions: (1) What proportion of the Ghanaian population had access to and had been vaccinated or willing to be vaccinated for the Coronavirus disease?; (2) To what extent did the COVID-19 Vaccine uptake or willingness to be vaccinated vary among the different population groups in Ghana?; (3) Which factors affected access to and uptake of the COVID-19 vaccines among eligible Ghanaian population?; and (4) What measures were taken or could have been taken to improve access to and uptake of the COVID-19 vaccines in Ghana?

## 2 Materials and methods

**Figure 1:**
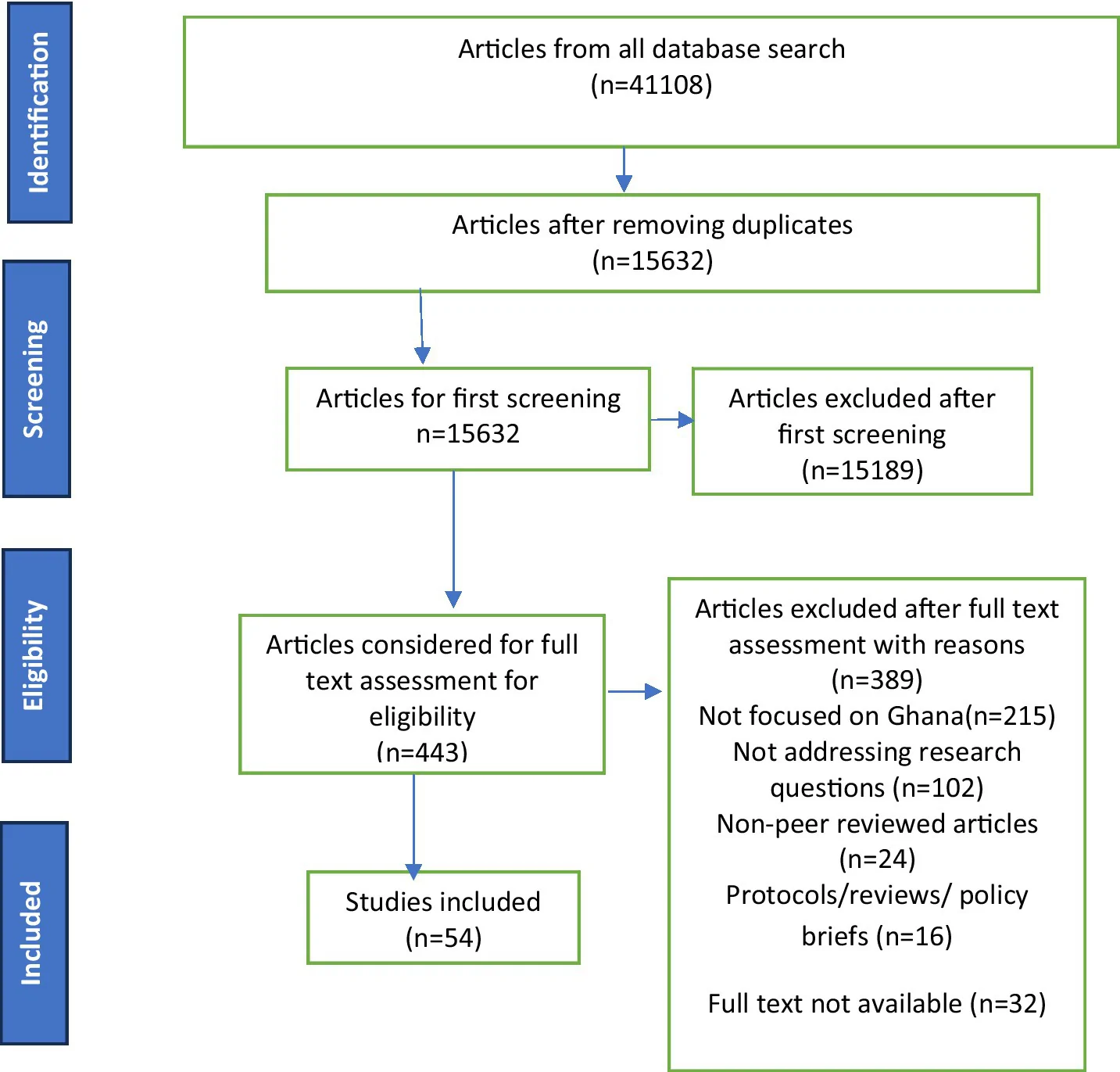
PRISMA flow chart.

We used a scoping review approach to explore what has already been documented on factors that have influenced access to and uptake of the COVID-19 vaccines and measures that can be taken to improve public health response strategy for future pandemics (21). The Arksey and O’Malley five stage analytic framework was adopted to facilitate the search for relevant literature (22) on the subject matter in Ghana. This framework provides a vivid and clear methodological approach and guidance on how the process can be replicated. The stages involved include: identifying the research questions (Stage 1); searching for relevant studies (Stage 2); selecting studies (Stage 3); charting the data (Stage 4); and collating, summarizing, and reporting the results (Stage 5). The Preferred Reporting Item for Systematic Reviews and Meta-Analyses extension for scoping reviews (PRISMA-ScR) was used throughout the entire review process and reporting. (see Figure 1-Study Selection Flow Chart) (23).

### 2.1 Search and identification of studies

We identified relevant literature for the study using electronic database searches such as PubMed, Cochrane, African journal online (AJOL), and Google Scholar. The search process retrieved published studies that had data on access to and uptake of COVID-19 vaccines; barriers to access and uptake and measures to improve access and uptake of COVID-19 vaccines in Ghana. We used snowballing to identify additional articles for consideration by screening through the references of already identified articles (reference mining).

Medical Subject Heading (MeSH), keywords, and free text search terms were used as the search terms. We included alternative terms for COVID-19 [MeSH Terms], combined them using Boolean operators search terms: (SARS) OR (CoV-2) OR (coronavirus) AND (Vaccination) OR (vaccine) OR (immunization) AND (Fair) OR (equitable) AND (timely) AND (patterns) AND (intake) OR (Uptake) OR (Use) AND (access) AND (barriers) OR (Hesitance) OR (obstacles) OR (disfavor) OR (dislike) AND (approach) AND (equitable) OR (Fair) AND (timely) OR (up-to-date) OR (disadvantaged) OR (underprivileged) OR (vulnerable groups) AND (Ghana).

### 2.2 Inclusion and exclusion criteria

Original research studies reporting access to and uptake of COVID-19 vaccines, barriers to access and uptake, and measures to improve access and uptake of COVID-19 vaccines were considered. We conducted title and abstract screening of the various studies retrieved to enable us identify studies that met all the elements for inclusion. The inclusion and exclusion criteria guided the selection process to determine which studies were most appropriate for full-text review and for inclusion in this review. These studies included cross-sectional and observational studies (case–control, and cohort), using qualitative, quantitative or mixed methods and reported between 2019 to early 2024. The first occurrence of the Coronavirus was the benchmark for the timeline consideration. Eligible studies met the following inclusion criteria of being; (1) peer-reviewed and published; (2) primarily discussing or evaluating access, uptake and hesitancy to COVID-19 vaccines; (3) studies that focused on Ghana; (4) published in English; (5) published between January 2020 to May 2024. On the other hand, editorial reports, letters, studies without full text, reviews, commentaries, policy briefs or protocols were all excluded.

Quality assessment was carried out using the Joanna Briggs Institute (JBI) (24) by two of the authors (JA and DA). The selection process followed through title and abstract screening, and full text reviews to facilitate the selection of appropriate studies for the review. This was aimed at improving rigor of the process and the narrative synthesis and discussion rather than a consideration for excluding studies of low quality.

### 2.3 Data extraction, processing, analysis and reporting

Data was charted using a data extraction sheet where summaries of relevant studies were collated. Relevant headings captured in the extraction sheet included: Authors and year of publication; the study setting; (i.e., country study was conducted and data collection period); study methodology; the main results and conclusion. The methodology section comprised of the study characteristics—(i.e., study design, target population, and sample size). The selection process is shown in the PRISMA flow diagram (Figure 1).

Even though this scoping review was not registered in any public platform (i.e., Open Science Framework (OSF) or Protocols.io), the protocol was developed and guided by the PRISMA-ScR and JBI methodology for scoping reviews.

For purposes of reporting the findings in this review, the data was categorized into themes and analyzed. The following specific categories were considered: (1) Acceptance and uptake of COVID-19 vaccines in Ghana; (2) COVID-19 vaccine uptake by demographic stratified groups; (3) Barriers to access and uptake of COVID-19 vaccines in Ghana; and (4) Measures to improve uptake of COVID-19 vaccines in Ghana. The researchers applied thematic narratives to report all data (5).

## 3 Results

Initial search from all databases produced a total of 41,138 records. After duplicates were removed, 15,632 articles were left for screening using the titles and abstracts to extract those that met the initial screening criteria. After this phase of screening, 15,189 articles were further excluded because they did not contain data that could help achieve the research objectives. Four hundred and forty-three (*n* = 443) articles were then considered for full-text review. Subjecting the remaining articles to the rigorous inclusion and exclusion process, 389 studies were excluded for various reasons (i.e., articles not peer reviewed, not focused on vaccine access/uptake; or hesitancy, not focused on Ghana, or the full text was not available). The remaining 54 articles were then included in the final analysis. The selection process is shown in the PRISMA flow diagram (Figure 1).

### 3.1 COVID-19 vaccines acceptance and uptake among the general population in Ghana

We explored access to and uptake/acceptance of the COVID-19 vaccines among the Ghanaian population. Table 1 summarizes studies that explored uptake or willingness to receive COVID-19 vaccines by the general Ghanaian population. We found that uptake or willingness to accept COVID-19 vaccines varied markedly among population type and demographic stratified groups. The highest COVID-19 vaccine uptake or willingness to receive vaccine by any group was 92.1% among health professionals as reported by Balegha et al. (10), while the lowest was 5% reported among the general adult population (25). Thirty-six articles were reviewed for vaccine uptake or willingness to receive vaccines, out of that, 17 articles (representing 47.2%) reported uptake/willingness to receive vaccines coverage of 70% and above, which is the standard requirement needed to generate sufficient herd immunity and reduce transmission of the coronavirus (26). On the other hand, 19 studies (representing 52.8%) out of the (*N* = 36) reviewed reported vaccine uptake/willingness to receive vaccine coverage below the 70% benchmark. Among all population groups that participated in the COVID-19 vaccination exercise, health professionals had the highest uptake rate in Ghana. Thus, 8 out of the 11 articles reviewed for vaccine uptake or willingness to receive vaccine among health professionals scored the 70% mark or more compared to the general population.

**Table 1:** COVID-19 vaccine uptake or acceptance among the general population.

Authors & year of publication	Country & data collection period	Methodology	Rate of uptake (%)
Nasiratu et al., 2023 (34)	Ghana October–November, 2022	Study design: Cross-sectional using structured questionnaire Population target: persons aged 18 years and above Sample size: 388	72.7% were willing to vaccinate if vaccines were made available; (66.2%) if vaccination was mandatory; (76.0%) when adequate information about the disease was given
Sampene et al., 2023 (25)	Ghana March–June 2021	Study design: Cross-sectional survey Population target: Persons 18 years and above Sample size: 400	Less than 5% of the participants had received vaccines
Afreh et al., 2023 (41)	Ghana, from 10 to 21 January 2022	Study design: Survey, with questionnaire on electronic devices Population target: Persons 18 years and above Sample size: 1,500	About1/3 of the eligible population was fully vaccinated; (46.7%) had received at least one COVID-19 vaccine dose, 69.4% of unvaccinated were willingness to receive the vaccine
Vepachedu et al., 2024 (68)	Ghana December 2021 and January 2022	Study design: Mobile phone-based surveys Population target: Persons 18 and above Sample size: 1,494	73% was vaccinated
Ankrah et al., 2021 (42)	Ghana—2nd March-19 May, 2021	Study design: Cross-sectional Population target: Health care workers Sample size: 240	66% two vaccinations, and almost 80% had at least one vaccination.
Atongu et al., 2024 (46)	Ghana, from 30 June to 15 August 2021	Study design: Cross-sectional Survey Population target: Health care workers Sample size: 128	72% had received the first dose
Atta-Osei et al., 2024 (6)	Ghana August and September 2021	Study design: Cross-sectional Survey Population target: Persons living with disability (PwDs) Sample size: 250	71.2% were willing to be vaccinated
Aram et al., 2022 (45)	Ghana January and March 2021	Study design: A cross-sectional online survey Population target: General population Sample size: 620	79% were willing to take a mandatory COVID-19 vaccine while 71% willing to take a voluntary shot
Alhassan et al., 2021 (43)	Ghana 18th September and 23rd October, 2020	Study design: Web-based cross-sectional survey Population target: Healthcare workers Sample size: 1,605	70% expressed willingness to accept the COVID-19 vaccine; 48% willing to participate in a COVID-19 vaccine trial
Alhassan et al., 2021 (43)	Ghana; from 18th September and 23rd October, 2020.	Study design: Online nation-wide survey Population target: all adults aged 18 years Sample size: 1,556	65% willing to accept COVID-19 vaccine
Alhassan et al., 2022 (44)	Ghana	Study design: A cross-sectional web-based survey Population target: Community members- adults 18 years and above. Sample size: 1,556	44.79% willing to receive the COVID-19 vaccine; Among those willing to accept the vaccine, 55% were willing to pay an average of US$6.00 for the vaccine
Amo-Adjei et al., 2022 (27)	Ghana April and May 2021	Study design: A sequential mixed-method investigation Population target: priority population: persons 60 years and above, frontline government functionaries, health workers, persons with underlying health conditions and, religious leaders and teachers. Sample size: 415	70% expressed willingness to take the vaccine; 20% will not accept
Amponsah-Tabi et al., 2023 (69)	Ghana-May to November, 2021	Study design: A cross-sectional study in 3 regions of Ghana Population target: Residents 15–81 years Sample size: 764	41.9% willing to accept COVID-19 vaccine
Lamptey et al., 2021 (38)	Ghana 14th October to the 12th of December 2020	Study design: A cross-sectional survey Population target: General Population Sample size: 1,000	54.1% were willing to accept COVID-19 vaccines
Aggrey-Bluwey & Abekah-Nkrumah, 2024 (70)	Ghana 5th and 23rd September 2022.	Study design: A qualitative-case study design, using in-depth interviews Population target: General population 18 + yrs. Sample size: 25	20% had been vaccinated against COVID-19
Adomako et al., 2021	Ghana 4th of April to 16th May, 2021.	Study design: online-based cross-sectional study Population target: General Population Sample size: 613	74.7% were willing to take the vaccines
Kyei-Arthur et al., 2022 (47)	Ghana over 3 months, ending November 2021	Study design: online Survey Population target: Parents and guardians Sample size: 415	73.3% of parents/guardians willing to allow their children to be vaccinated against COVID-19
Agyekum et al., 2021 (40)	Ghana; from 16 January to 15 February 2021	Study design: cross-sectional online survey Population target: Health workers Sample size: 234	f39.3% were willing to be vaccinated
Nanteer-Oteng et al., 2022 (55)	Ghana	Study design: cross-sectional survey using snowball and convenience sampling Population target: Persons 18 years and above Sample size: 492	67.5% had been vaccinated;
Mbele et al., 2024 (71)	Ghana; 24th July 2023 to 31st August 2023	Study design: cross-sectional in a post vaccine roll out period Population target: Healthcare workers Sample size: 256	85.9% had received at least one dose of the COVID-19 vaccination
Botwe et al., 2022 (8)	Ghana; 24th–28th February 2022.	Study design: cross-sectional survey using quantitative methods Population target: Healthcare workers Sample size: 108	59.3% of participants were willing to take the vaccine
Asumah et al., 2022 (28)	Ghana; 10 weeks, from January 2021 to March 2021.	Study design: cross-sectional using quantitative approach Population target: Healthcare workers Sample size: 215	78.6% overall vaccine acceptance rate
Okai & Abeka-Nkrumah, 2022 (60)	Ghana; 18th May 2021 to 14th July 2021	Study design: a cross-sectional online survey. Population target: adults 18 years and above Sample size: 362	62.7% willing to be vaccinated
Serwaa et al., 2021 (37)	Ghana 14th October–12th December, 2020	Study design: cross-sectional survey. Population target: General population Sample size: 1,000	54.1% Willing to be vaccinated
Amoah et al., 2024 (30)	Ghana	Study design: A quantitative research approach Population target: General population Sample size: 408 individuals from 204 households	41% were completely vaccinated while 59% were under vaccinated
Yeboah et al., 2021 (59)	Ghana; from September 2020 to December 2020.	Study design: a cross-sectional study employing an interview-structured questionnaire. Population target: General population Sample size: 1,560	35.3% were willing to be vaccinated.
Mohammed et al. 2023 (7)	Ghana; from September 2020 to December 2020.	Study design: A hospital-based cross-sectional study with focus on quantitative approach. Population target: Health workers Sample size: 424	73.6% was vaccinated Among those who did not take the vaccine, 64.3% were willing to take it in the future.
Balegha et al., 2024 (10)	Ghana; from January 16th to February 28th	Study design: Cross-sectional study using a multi-centre E-survey. Population target: Health professionals Sample size: 403	92.1% had been vaccinated with Oxford AstraZeneca vaccines
Annan et al., 2021 (72)	Ghana; from January 16th to February 28th	Study design: cross-sectional survey Population target: Health professionals-Junior doctors Sample size: 305	66.9% willing to take the vaccine when available.
Dubik et al., 2022 (73)	Ghana; from 18th May to 14th July 2021	Study design: Cross-sectional online survey Population target: Ghanaian adults (18 years and above) Sample size: 362	62.7% willing to take vaccines, the decision to accept the COVID-19 vaccine was influenced by occupation, perceived susceptibility, perceived benefits and attitudes towards the vaccines.
Forkuo et al., 2022 (31)	Ghana; from 18th May to 14th July 2021	Study design: analytical cross-sectional study Population target: Ghanaian adults (18 years and above) Sample size: 325	9.8% had been vaccinated. While 82.6% indicated COVID 19 vaccine acceptance among the unvaccinated. Major reason for vaccine acceptance was “it could protect against COVID-19” (96.7%)
Owusu et al., 2024 (74)	Ghana; from 18th May to 14th July 2021	Study design: Online survey Population target: Nurses & midwife students Sample size: 557	80% were vaccinated.
Bobie et al., 2022 (75)	Ghana; January and March 2021	Study design: A quantitative cross-sectional study Population target: Persons 18 years and above Sample size: 267	41.95% willing to accept the COVID-19 vaccine.
Udor et al., 2023 (35)	Ghana; from 22nd March 2021 to 15th April 2021	Study design: Online survey Population target: Adult Ghanaians Sample size: 331	5.8% had been vaccinated, among those who had not been vaccinated, 54.3% were willing to be vaccinated.
Morgan et al., 2023 (33)	Ghana; between June 2021 and August 2021.	Study design: A cross-sectional survey with a quantitative approach. Population target: Older adults (aged 50 years and older) Sample size: 400	5% had been vaccinated, with 79% willingness to be vaccinated.
Seidu et al., 2024 (29)	Ghana; from December 2021 to March 2022	Study design: A cross‐sectional survey Population target: Persons living with disabilities (PwDs) Sample size: 402	68.7% had received the COVID‐19 vaccine.

We reviewed only two studies that reported vaccine uptake among the vulnerable population in Ghana. For the purpose of this analysis, the vulnerable population was identified to include persons living with any form of disability; persons aged 60+ years; persons who already have some health challenges; health workers at the forefront of the vaccination exercise, and others identified as vulnerable by the authors. We found that COVID-19 vaccine uptake among this group was relatively high (70+%) (6, 27), probably because of the general belief that this population group is already susceptible to diseases by their unique disposition. Amo-Adjei and colleagues emphasized that vulnerable population were more likely to accept the COVID-19 vaccine if they trusted in the efficacy and safety of the vaccines (27). Asumah et al., on the other hand noted that among this vulnerable population, vaccine uptake was largely impacted by those who had already taken the vaccines without any problems; people who had never refused any vaccines in their lifetime, and those who held the view that the COVID-19 vaccines in Ghana were safe and effective in controlling the Coronavirus transmissions. Other factors observed included the fact that those who sought advice from health professional prior to taking the vaccines; and those who felt it was convenient to take the vaccines were more likely to participate in the COVID-19 vaccination exercise (28) (see Table 1).

### 3.2 COVID-19 vaccine acceptance or uptake by demographic stratified groups in Ghana

We analyzed twenty-six studies (*N* = 26) for vaccine uptake or acceptance by demographic variables. The findings suggest that age, marital status, gender, and religion correlated with the COVID-19 vaccine uptake. Thus, five studies (*n* = 5) out of nine, and eight (*n* = 8) out of 11 assessed marital status and age, respectively, for vaccine uptake. We found that older people (6, 25, 29–33) and being single (32, 34–37) had higher chances of COVID-19 vaccine acceptance or uptake compared to younger people and those who were married. Similarly, being a male (34, 35, 37–44) and a Muslims (30, 41, 43, 45) had higher propensity for vaccine acceptance compared to being a female and a Christian as reported in 11 (*n* = 11) out of 15 and four (*n* = 4) out of 7 studies reviewed for gender and religion, respectively. Exploring the level of education and its association with the COVID-19 vaccine uptake, we found that higher educational attainment correlated more with vaccine uptake (6, 29, 35, 36, 43, 46) than people with lower levels of education (25, 41, 47), (see Table 2 and frequency tables).

**Table 2:** COVID-19 vaccine uptake or acceptance stratified by demographic groups.

Authors & year of publication	Country & data collection period	Methodology	Uptake by demographic stratification
Seidu et al., 2024 (29)	Ghana; from December 2021 to March 2022	Study design: A cross‐sectional survey Population target: Persons living with disabilities (PwDs) Sample size: 402	Vaccine uptake was higher among PWDs with visual impairments, older age groups (60 and above), those with junior high school level of education, and those who were employed, compared to their respective reference groups.
Amponsah-Tabi et al., 2023 (69)	Ghana-May to November, 2021	Study design: A cross-sectional study in 3 regions of Ghana Population target: Residents 15–81 years Sample size: 764	educational level, employment status were significantly associated with COVID-19 vaccine acceptability;
Atongu et al., 2024 (46)	Ghana, from 30 June to 15 August 2021	Study design: Cross-sectional Survey Population target: Health care workers Sample size: 128	Residence and attaining higher education were significantly associated with Covishield uptake as was attitude; Urban residents had over five times higher odds of vaccine uptake than rural residents.
Lamptey et al., 2021 (38)	Ghana 14th October to the 12th of December 2020	Study design: A cross-sectional survey Population target: General Population Sample size: 1000	Participants who were 36–45 years of age had lower odds of accepting the vaccine if available compared to those aged 18–25 years Participants aged 26–35 years (71.7%), who were males (60.5%) and were single (58.3%) intended to accept the vaccine; Accepting a potential vaccine was lower in females relative to males; Married participants were more likely to accept the vaccine compared to the singles; Government workers were more likely to accepting the vaccine
Nasiratu et al., 2023 (34)	Ghana October–November, 2022	Study design: Cross-sectional using structured questionnaire Population target: persons aged 18 years and above Sample size: 388	Vaccine uptake was higher among men than women; Single respondents had 2 times the odds of receiving COVID-19 vaccines compared to those who were married; Muslims were less likely to receive COVID-19 vaccination than the Christians
Sampene et al., 2023 (25)	Ghana March–June 2021	Study design: Cross-sectional survey Population target: Persons 18 years and above Sample size: 400	participants between the ages of 25 and 35 had a higher percentage of refusing the vaccine; Persons with university educational had the highest vaccine refusal rate; Single or unmarried participants also had a greater odds ratio of acceptors with doubts than those married
Bobie et al., 2022 (75)	Ghana; January and March 2021	Study design: A quantitative cross-sectional study Population target: Persons 18 years and above Sample size: 267	female respondents were more likely to accept the COVID-19 vaccine compared to their male counterparts;
Udor et al., 2023 (35)	Ghana 22nd March 2021 and 15th April 2021	Study design: online survey Population target: general population Sample size: 331	About half of the males and less than half of the females had a positive intention for the vaccine; More than half of the respondents with doctorate degrees and those who were neither married nor in a union showed interest in the COVID-19 vaccine
Yorke et al., 2023 (36)	Ghana October and November 2020.	Study design: cross-sectional population-based study. Population target: All adult residents in the communities 18 years and above Sample size: 997	As the educational level increases, the mean score for vaccine acceptability also increases; Unmarried participants had a higher mean score of vaccine acceptability than married participants; unemployed participants had the highest vaccine acceptability score
Awuni et al., 2022 (39)	Ghana; from 16th to 20th April, 2021	Study design: Cross-sectional using questionnaire Population target: literate Ghanaians-general population Sample size: 223	More males (47.71%) indicated willingness to take the vaccine without hesitation when it was made available to them compared to females (31.43%)
Atta-Osei et al., 2024 (6)	Ghana August and September 2021	Study design: Cross-sectional Survey Population target: Persons living with disability Sample size: 250	Age, religion, knowledge of COVID-19, and educational level were contributing factors to their willingness to accept the COVID-19 vaccine; Older people and those with higher education were more likely to accept the vaccine compared to younger people and those with no or less education
Adjaottor et al., 2022 (76)	Ghana; A single day was used to collect the data in each of the study sites	Study design: Cross-sectional design study Population target: Adult Ghanaians Sample size: 817	Females believed COVID-19 information and accepted COVID-19 vaccination more than males did
Kyei-Arthur et al., 2022 (47)	Ghana over 3 months, ending November 2021	Study design: online Survey Population target: Parents and guardians Sample size: 415	Parents and guardians with SHS education were less likely to accept the COVID-19 vaccine for their children than those with less than Senior High School
Agyekum et al., 2021 (40)	Ghana; from 16 January to 15 February 2021	Study design: cross-sectional online survey Population target: Health workers Sample size: 234	Higher proportion of females (66.9%) significantly indicated non-acceptance of COVID-19 vaccine compared to males; A higher proportion of those who were never married (69.3%) indicated non-acceptance of the COVID-19 vaccine compared to those who were married; Female health care workers were less likely to accept COVID-19 vaccine than males.
Alhassan et al., 2021 (43)	Ghana 18th September to 23rd October, 2020	Study design: nation-wide survey Population target: Community members- adults aged 18–48 years Sample size: 1,556	Females were 11 times less likely to accept a COVID-19 vaccine when given the opportunity compared to males
Aram et al., 2022 (45)	Ghana January and March 2021	Study design: A cross-sectional online survey Population target: General population Sample size: 620	Muslims were more likely to take a mandatory COVID-19 vaccine shot as compared to Christians; Ghanaians who were above 50 years were more likely to take a mandatory COVID-19 vaccine shot as compared to their 18–24 years counterparts
Serwaa et al., 2021 (37)	Ghana 14th October–12th December, 2020	Study design: cross-sectional survey. Population target: General population Sample size: 1,000	Participants aged 26–35 years 277 (71.7%), who were males 201 (60.5%) and were single 413 (58.3%) intended to accept the vaccine.
Amoah et al., 2024 (30)	Ghana	Study design: A quantitative research approach Population target: General population Sample size: 408 individuals from 204 households	Under-vaccination was significantly high (72.32%) among respondents aged 20–29. The results revealed that uptake of the COVID vaccine significantly varied by religion: Muslims (48.70%) and Christians (38.25%), with approximately 10% more Muslims reporting being vaccinated compared to Christians.
Adomako et al., 2021	Ghana; from 4th of April to 16th May, 2021	Study design: cross-sectional -online survey Population target: General population Sample size: 613	Young people, females, and people with higher education were reported not likely to accept the vaccines if they were made available.
Annan et al., 2021 (72)	Ghana; from January 16th to February 28th	Study design: cross-sectional survey Population target: Health professionals-Junior doctors Sample size: 305	Females, young people (25–30 yrs), and Christian were more likely to take the vaccine
Afreh et al., 2023 (41)	Ghana; from 10 to 21 January, 2022.	Study design: cross-sectional -survey using electronic devices Population target: General population Sample size: 1,500	High levels of mistrust, being female, greater years of education, and being Christian, were key predictors to vaccine hesitancy
Forkuo et al., 2022 (31)	Ghana; from 18th May to 14th July 2021	Study design: analytical cross-sectional study Population target: Ghanaian adults (18 years and above) Sample size: 325	Older people and those married or widowed were more likely to receive the vaccines compared to younger folks and singles
Ankrah et al., 2021 (42)	Ghana—2nd March–19 May, 2021	Study design: Cross-sectional Population target: Health care workers Sample size: 240	Although males were more likely to vaccinate compared to females, this was not significant.
Alhassan et al., 2021 (43)	Ghana; from 18th September to 23rd October, 2020.	Study design: Online survey across all 16 administrative regions of Ghana. Population target: all adults aged 18 years Sample size: 1,556	Willingness to voluntarily participate in COVID‑19 vaccine trial, uptake the vaccine and advise others to do same was higher among adults aged 18–48 years, the unmarried and males (*p* < 0.05).
Alhassan et al., 2021 (43)	Ghana 18th September and 23rd October, 2020	Study design: web-based cross-sectional survey Population target: Healthcare workers in all 16 administrative regions Sample size: 1,605	Younger HCWs, non‑Christians and those who worked in faith‑based health facilities were more likely to participate in a COVID‑19 vaccine trial. Female HCWs and those with lower educational qualification were less likely to accept a COVID‑19 vaccine
Morgan et al., 2023 (33)	Ghana; between June 2021 and August 2021.	Study design: A cross-sectional survey with a quantitative approach. Population target: Older adults (aged 50 years and older) Sample size: 400	Females and those who have retired were significantly less likely to engage in COVID-19 vaccine hesitance; participants who trust public health information and have social capital were significantly less likely to present COVID-19 vaccine hesitance

### 3.3 Barriers to acceptance and uptake of COVID-19 vaccines in Ghana

In this section, we explored barriers that limited people from accessing or receiving the COVID-19 vaccines even if they had intentions and were willing to do so. Eighteen (*N* = 18) studies reported factors that facilitated or served as barriers to the uptake of COVID-19 vaccines in Ghana. For the purpose of this report, we categorized these factors into three main groups as below.

#### 3.3.1 Perceived side effects and safety concerns about the COVID-19 vaccines

Safety concerns and perceived side effects of the COVID-19 vaccines emerged as a major factor that affected successful implementation of the COVID-19 vaccine campaign and uptake in Ghana. Thirteen studies (*n* = 13) representing (72.2%) reported that some Ghanaians who expressed concerns about the safety of the vaccines or had fears of side effects or had heard about side effects experienced by others who took the vaccines had reservations in participating in the vaccination exercise (8, 25, 31–32, 35, 40, 41, 47–51). Among some of the fears cited included feeling feverish; joint pains; pains or swelling at point of injection; slight body pains; malaria symptoms within 24 h after injection; fatigue; headaches and muscle pain; and general weakness as noted by Dovie et al. (50).

#### 3.3.2 Health system factors

We found that health system factors such as long distance to vaccination Centres, long queues at vaccination Centres, inadequate vaccinators, stock-outs, and unavailability of vaccinators at designated Centres and general systemic challenges affected vaccine uptake. Thus, (*n* = 12) studies representing (66.7%) of studies reviewed for factors affecting vaccine uptake cited health system factors to have affected uptake (8, 25, 27, 32, 35, 38, 41, 49–52). Lamptey and colleagues observed that participants who mistrusted the health system were unwilling to receive the COVID-19 vaccine even if they had access to them. On the other hand, Abraham et al. indicated that dissatisfaction among the COVID-19 vaccinators over delayed allowances, shortfalls in logistics arrangement including transportation and venues for the vaccination exercise affected vaccine uptake. The study further identified challenges in data capture and retrieval due to unstable internet access as serious setbacks in the vaccination campaign.

Equally, shortfalls in logistics and proximity to the vaccination centers served as a barrier to vaccine uptake. Afrifa-Anane et al. disclosed that long hours of waiting to receive the vaccines at the vaccination centres, and inadequate supply of vaccines were significant barriers to the COVID-19 vaccination exercise in Ghana. It was also reported that, people who lived in hard-to reach areas had difficulty getting access to the vaccines as it was increasingly difficult to get to some communities in very remote areas (53).

#### 3.3.3 Conspiracy theories, myths, misconceptions and religious beliefs

Myths, misconceptions, socio-cultural and religious factors, and conspiracy theories were reported to have served as one of the critical bottlenecks to the COVID-19 vaccination campaign. Ten (*n* = 10) studies constituting 55.6% of studies reviewed for factors affecting vaccine uptake reported this (8, 33, 35, 49, 50, 52, 54). For example, Aberese-Ako et al. (33, 54) reported that conspiracy theories, suggesting that the vaccines had the tendency to reduce lifespan, eliminate Africans, make people foolish and cause infertility in those who got vaccinated, or viewed as a means of subscribing to the “devil,” affected uptake. Similarly, Afrifa-Anane et al. reported religious persuasions and misconceptions such as vaccine being cause of impotency, bareness and making people foolish as factors hindering vaccine uptake. They indicated that some women refused to take the COVID-19 vaccines because they viewed it as emanating from the devil as admonished by their spiritual guardians (49).

#### 3.3.4 Other barriers

Various media platforms including social media were reported to have contributed to building perceptions which were critical in decision making regarding the acceptance and uptake of the COVID-19 vaccines in Ghana. Dovie et al. (50) revealed that negative reporting through the various social media platforms about the Coronavirus and the COVID-19 vaccines about the role of government in the vaccine campaign created mistrust among the populace and affected acceptance and uptake.

Others reported that some Ghanaians believed the COVID-19 vaccines were not efficacious because they were given to them for free. There were also, those who linked the countries where the vaccines were made to their effectiveness. For example, Johnson and Johnson which were produced in the United States of America (USA) were perceived to be more effective than AstraZeneca from India. The study further posited that pregnant women were not likely to be vaccinated once they were not certain about the effect of the vaccine on their unborn babies (49) (see frequency tables for factors affecting vaccine uptake) (Tables 3–6).

**Table 3:** Frequency table for COVID-19 vaccine uptake or acceptance in Ghana.

Indicator	Studies covered	Studies that cited	Percentage
Vaccine uptake or acceptance among the general population			
Vaccine uptake/ willingness to receive vaccines coverage ≥70%	36	17	47.2%
Vaccine uptake/willingness to receive vaccine coverage <70%	36	19	52.8%
Vaccine uptake or willingness to receive vaccine coverage ≥70% among health professionals	11	8	72.7%
Vaccine uptake or willingness to receive vaccine coverage <70% among health professionals	11	3	27.3
Vaccine uptake or willingness to receive vaccine coverage among vulnerable population ≥70%	2	2	100%

**Table 4:** Frequency table for COVID-19 vaccine uptake or acceptance by demographic stratified groups.

Indicator	Studies covered	Studies cited (%)	
Total number of articles reviewed (*N* = 26)			
Vaccine Uptake or Acceptance by Age	11	Older (*n* = 8); (72.7%)	Younger (*n* = 3); (27.3%)
Marital status and vaccine uptake	9	Married (*n* = 4); (44.4%)	Single (*n* = 5); (55.6%)
Vaccine Uptake or Acceptance by Gender	15	Male (*n* = 11); (73.3%)	Female (*n* = 4); (26.7%)
Vaccine Uptake or Acceptance by Religion	7	Muslim (*n* = 4); (57.1%)	Christian (*n* = 3); (42.9%)
Vaccine Uptake or Acceptance by level Education	10	Higher educational attainment (*n* = 6); (60%)	Lower level of education (*n* = 4); (40%)

**Table 5:** Frequency summary table: factors affecting vaccine uptake and acceptance.

Indicator	Studies covered	Studies cited	Percentage
Safety concerns and fear of side effects	18	13	72.2%
Individual and Health system factors (e.g long distance to vaccination Centres, long queues, inadequate vaccinators, not susceptible to Covid-19, did not need the vaccines, tight work schedules, belief that there are better ways to stop virus spread etc)	18	12	66.7%
Conspiracy theory, Myths, misconceptions & misinformation and religious factors	18	10	55.6%

**Table 6:** Frequency table: factors promoting vaccine uptake and acceptance.

Indicator	Studies covered	Studies cited	Percentage
Fear of side effects	18	12	66.7%
Individual factors	18	7	38.9%
Conspiracy theory	18	6	33.3%
Safety concerns	18	6	33.3%
Health system factors and political influence	18	6	33.3%
Religious and cultural beliefs	18	5	27.8%
Myths, misconceptions & misinformation	18	4	22.2%
Vaccine stock-outs	18	4	22.2%
Long distance to vaccination centres	18	2	11.1%
Doubt about effectiveness and efficiency of vaccines	18	2	11.1%

### 3.4 Measures to improve acceptance and uptake of COVID-19 vaccines in Ghana

Improving acceptance and uptake of vaccines required that factors that inhibited access and uptake of the vaccines are addressed. This included the need to tackle doubts about the safety and efficacy of vaccines, socio-cultural and religious factors, trust issues and conspiracy theories around particularly the Coronavirus and the COVID-19 vaccines.

Almost all the articles reviewed suggested public health education and promotional activities as measures that could dispel doubts about the disease and the vaccines and create a positive image that could propel acceptance and intake of the vaccines. In this regard, most of the researchers suggested the need to intensify public health education and health promotion activities using innovative and diverse methods targeted at addressing safety concerns and perceived side effects that confront vaccine uptake (11, 52, 55–57).

For public education strategies to be effective, some recommended the need to take into consideration the geographic and demographic differences of the communities so as to meet the needs of the different age groups and geographic settings. Also, since the issue of trust about the vaccines was a major setback in the COVID-19 vaccine campaign exercise, effective engagement and collaboration with community leaders, including religious and traditional leaders, was recommended to play a critical role in public educational campaign strategies (19, 35, 58, 59). Morgan et al., have also suggested a multisectoral stakeholder consultation and collaboration within the health sector and beyond to design interventions that will increase public education and raise awareness for all groups (33).

Furthermore, to address issues related to negative reportage and perceptions about the Coronavirus and the COVID-19 Vaccines, Okai et al. suggested that vaccine campaign messages should be tailored towards addressing issues of vaccine safety and efficacy to improve and sustain vaccine uptake towards achieving herd immunity (60). In line with this, Awuni et al. emphasized the need for vaccine advocacy groups and campaigners to focus campaign messages on the safety and efficacy of the specific vaccines approved for use (39). They concluded that this has the tendency to dispel negative perceptions about the vaccines and promote vaccine acceptance in the country.

In addition, it was suggested that future vaccine campaign efforts should target specifically the vulnerable population including women who are disproportionately affected by the impact of the Coronavirus pandemic (39). In view of public health education in respect of the broad spectrum of the vulnerable population, some have proposed inclusive and targeted educational campaign strategy including house to house campaigns for the aged and people living with disabilities and comorbidities to dispel misconceptions around their peculiar conditions and vaccines uptake and its repercussions for the future (6, 15, 29, 61). They concluded that future vaccine campaign strategies should prioritize this group to get the best outcomes from any vaccination exercise.

In order to address conspiracy theories, misinformation and disinformation around the Coronavirus and COVID-19 vaccines, some studies have recommended that credible institutions and stakeholders should lead the public health communication campaigns to educate the public as this would serve as credible and reliable sources of COVID-19 pandemic information (15, 57, 62). This should aim at increasing communication and sensitization towards improving COVID-19 vaccine coverage in Ghana. Regarding those who expressed doubts about the existence of the Coronavirus and its effect in Ghana, some studies have stated the need to make public detailed information about those affected by the Coronavirus pandemic. This information should spell out in disaggregated form details of people who have contracted the virus, those who have died from the virus, facilities where they were admitted or died so as to erase public suspicion about the disease as a fallacy and boost vaccine uptake (54).

For the health system barriers, early and adequate education on vaccination in the facilities and during community outreach clinics is recommended to improve community perception about the potential adverse impact of the disease and engender acceptability. Furthermore, health sector players including the Ministry of Health, health service providers, and other stakeholders should play an active role in providing logistical support, ensuring access to vaccines, and managing vaccine campaign programs to improve vaccine acceptability and uptake (30, 55).

## 4 Discussion

The Coronavirus pandemic has brought a lot of hardship particularly to low-income households in developing countries (63). This has necessitated an increase in vaccination coverages in a sustained manner in these regions to contain the spread of the pandemic and provide protection to the populace. The analysis, however, showed a wide variation in vaccine intention and uptake among the general population and also demographic stratified groups. Health care workers were identified as the population group with the highest uptake rate followed by people identified to fall within the vulnerable population group. Even though several studies (47.2%) reported COVID-19 vaccine intension or uptake rate of 70%+ which is within the WHO’s envisioned threshold (26), more than half of studies reviewed (52.8%) reported uptake or willingness to receive vaccines below the WHO threshold. This is inconsistent with the work of Kanyanda et al. (4), who reported acceptance rate of COVID-19 vaccines with at least 4 in 5 people willing to be vaccinated in all six countries surveyed in sub-Sahara Africa except one. Similarly, Ghana’s performance in the COVID-19 vaccination campaign seemed to be at the same level with neighboring countries like Burkina Faso (74.4%) and Nigeria (78.4%) but below other African countries like Uganda (90.8%) and Kenya (95.1%) as reported by Wollburg et al. (64). Even though the COVID-19 vaccines coverage appeared relatively high in Ghana, there is still the need to strengthen Ghana’s response strategy to pandemics through collaborative effort to achieve global targets.

Despite the fact that Ghana’s strategy to get its population vaccinated in line with global set goals achieved some success, misconception about the Coronavirus and the COVID-19 vaccines thwarted this effort. Thus, the issue of misconceptions about the vaccines must be given serious attention and measures taken to address them as they could potentially disrupt future vaccine campaigns and derail the success chalked in Ghana’s Expanded Programme on Immunization (EPI) (65). Misconceptions and misinformation emanating from conspiracy theory have become critical avenue for distorting any vaccine campaign strategy and particularly in the African setting where religion and customary practices are shown to have influenced decision making towards the vaccines (49, 57). In the light of this, our findings documented measures that can be taken to ameliorate the impact of misconceptions and misinformation towards improving vaccine uptake coverage in Ghana.

One of the measures recommended is the inclusion of traditional, religious and community leaders in the execution of the vaccine campaign strategies. This approach is very much in line with Afolabi et al. (66) and Hopkins et al. (67) who recommended community involvement using local leaders, health workers and civil society organizations to disseminate health education and promotion information relative to promoting participation in health programs including vaccines. The use of feedback mechanisms is also an important component of the community-based approach which takes into consideration community support in health programs to encourage COVID-19 vaccine uptake. We, however, suggest that such community led approach should not only rely on awareness creation about the virus and the vaccines but also emphasize the impact the disease could have in the lives of the people. For instance, while Sampene et al. reported that about 92.75% of research participants had heard about Coronavirus, only about 5% of the participants had received the vaccines (25). This suggest that even though people may have good knowledge about the COVID-19 virus, that may not necessarily translate into acceptance and uptake. Other factors such as logistical challenges, socio-cultural, religious, geographic or even personal idiosyncrasies could serve as a barrier to vaccine uptake. This, therefore, requires targeted interventions to get people to understand the impact of taking the vaccines particularly for the vulnerable population and individuals with special needs to achieve herd immunity.

On the other hand, multistakeholder collaboration that brings together all the relevant stakeholders in the vaccine procurement, delivery and distribution chain, to the final consumer is needed for addressing the health system challenges that affect access and uptake of vaccines. This collaboration if properly harnessed, can ensure availability, accessibility and acceptability of vaccines by people who need them at the right time and at the right places.

The review did not find any significant variations in the uptake of the COVID-19 vaccines over the period (i.e., uptake was expected to improve over time), that the studies were conducted and reported. This may seem to suggest that the interventions that may have been put in place to boost uptake may not have made the desired impact. Secondly, it could also be attributed to hesitancy factors or even shortfalls or non-availability of the vaccines as played out by vaccine nationalism by developed countries. This would, therefore, require a reassessment and re-evaluation of the strategies that may have been put in place by the Ministry of Health and the Ghana Health Services and allied agencies to plan more effectively for future interventions.

## 5 Strengths and limitations

The use of the mixed method approach involving both quantitative and qualitative enabled the researchers to explore the proportions of the population that participated in the COVID-19 vaccination exercises and those that did not, and the reasons why people did not participate in the exercises. This brings out the nuances and effective planning for future vaccine preventable pandemics. The limitations of the study however, is that only peer reviewed articles were used for the analysis- other reports could have given different dimensions to the issues explored. The qualitative aspect did not also explore the reasons that influenced people’s participation in the vaccination exercise. This means that further analysis is needed to explore the factors that precipitated vaccine uptake among all populations.

## 6 Conclusion

The findings suggest that, even though Ghana’s effort to increase vaccine coverage across the general population and demographically stratified groups may have achieved some success, it was still far from meeting the global targets at achieving herd immunity and reducing the impact of the Coronavirus pandemic. Many factors including limited vaccine supply at the early stages; weak health system capacity and workforce to store and move vaccines across different geographic locations and population groups; hesitancy factors borne out of misconception and misinformation, myths and socio-cultural and religious beliefs about the virus and the vaccines and weak public health preparedness and response strategy may have derailed Ghana’s effort at improving access and uptake in meeting global targets in the fight against the coronavirus. These factors could also have a cascading effect on the gains Ghana has made over the years through the EPI programme and the overall vaccine campaign program in reducing morbidities and mortalities and improving population health, if not properly addressed. Addressing these, will require a sustained health system strengthening through multi-stakeholder collaboration and community-based engagement that would engender an effective public health preparedness and response strategy and immunization program towards improving access to and uptake of vaccines, and particularly in addressing future vaccine preventable pandemics in Ghana.

## Data Availability

The original contributions presented in the study are included in the article/supplementary material, further inquiries can be directed to the corresponding author.

## Glossary

AfHEA: African Health Economics and Policy Association
AJOL: African journal online
COVAX: COVID-19 Vaccine Global Access
COVID-19: Coronavirus 2019 Disease
CSO: Civil Society Organization
CVV: Coronavirus vaccine
ECOVA: Equitable Access to the COVID-19 Vaccines in Africa
EPI: Expanded Programme on Immunization
GHS: Ghana Health Service
HCW: Health Care Worker
HIC: High Income Countries
IDRC: International Development Research Centre
ISD: Information Services Department
JBI: Joanna Briggs Institute
LMIC: Low- and Middle-Income Countries
MeSH: Medical Subject Heading
MOH: Ministry of Health
NCCE: National Commission for Civic Education
PwD: Persons living with Disabilities
PRISMA: Preferred Reporting Items for Systematic Reviews and Meta-Analyses
TV: Television
USA: United States of America
WHO: World Health Organization
