# Correction: Factors affecting equitable access and uptake of COVID-19 vaccines in Ghana: a scoping review

**DOI:** 10.3389/fpubh.2026.1974927

**Published:** 2026-09-01

**Authors:** Frontiers Production Office

**Affiliations:** Frontiers Media SA, Lausanne, Switzerland

**Keywords:** coronavirus, vaccination, vaccine, equitable, access, uptake, scoping review, Ghana

The **Funding** of the article processing charges was erroneously omitted. The following text has now been added to the **Funding** statement:

“The article processing charges for this article were funded by the International Development Research Centre (IDRC). The funder had no involvement in the handling, peer review, or editorial decision for this article.”

The previous sentence in the Funding statement “The manuscript specifically did not receive any funding, while the overall project's funders had no role in determining the study's outcomes.” has been removed.

The full **Funding** statement will now read:

“The author(s) declared that financial support was received for this work and/or its publication. This research project under which this manuscript was written was funded by the International Development Research Centre (IDRC; Grant no: 109737-001). The article processing charges for this article were funded by the International Development Research Centre (IDRC). The funder had no involvement in the handling, peer review, or editorial decision for this article.

The original version of this article has been updated.

